# Managing major incidents 2012

**DOI:** 10.1186/1757-7241-21-S1-A7

**Published:** 2013-05-28

**Authors:** F Bird, N Pritchard

**Affiliations:** 1 Bartshealth NHS Trust, UK

## 

There were six presentations on the management of major incidents. The scene was set with a detailed account of a recent high profile event. The clinical aspects of blood in pre-hospital care followed and the last presentations concentrated n practical aspects of rescuer safety.

## Lessons from the Otoya Shootings and Oslo Bombing

Dr Solid, a Consultant in the Norwegian Ambulance and Dean of the Norwegian Air Ambulance Academy gave the keynote address; a candid and moving personal account of the tragic events that affected Norway in July 2011, claiming 77 lives. He described the difficulties faced and lessons learnt from dealing with the two sequential terrorist attacks; the bombing in Oslo and shootings on Utoya Island. Oslo saw in excess of 70 ambulances dispatched and 4 HEMS crews. He described the difficulties in establishing triage points, with the novel use of buses in Oslo as a holding point for casualties with minor injuries. He spoke of the challenges of working in an unpredictable, high-pressured environment without adequate protective measures available for those providing medical aid. He identified the familiar constraints of a ongoing incident, namely limited resources (notably stretchers) and intermittent unpredictable communication (whilst advocating the Tetra system that was used on Utoya Island). Dr Solid recognised the essential role of HEMS as a transport resource, the need for a closed air space at the earliest opportunity, and the benefit of a national response protocol to any anticipated major incident.

## Coagulation coagulopathy in pre hospital care and blood in majors incidents

The afternoon heard two complementary talks given by Mr Ross Davenport and Dr Simon Glasgow about trauma induced coagulopathy, and the use of blood products in major incidents, respectively. Dr Davenport described recent advances in the understanding of the coagulopathic state seen in trauma casualties, including an upregulation in activated protein C, fibrinolysis and deficiency in clotting factor V. Given the increase in mortality associated with an INR > 1.2, he advocated the use of point of care testing as a diagnostic tool (i.e. ROTEM, TEG), but highlighted its’ limitations particularly in patients with a low haematocrit. In the management of the coagulopahtic trauma victim, there was a resounding recommendation from both speakers for the use of a 1:1 ratio of packed red blood cells (PRBC) : fresh frozen plasma (FFP), but recognition of the difficulty in providing this given the current absence of available thawed FFP in the pre hospital setting in the UK. Other recommendations highlighted by Mr Davenport included the use of tranaxemaic acid, a Code Red protocol (as used in London Major Trauma Centres), early administration of other clotting factors, and the potential role of prothrombin complex concentrate (PCC) and fibrinogen concentrate in the reversal of Warfarin in head injury and trauma patients. He discussed to the potential future developments in the role of cryoprecipitate in major traumatic haemorrhage and the use of freeze dried platelets in a pre hospital setting. Following a brief history in the use of blood products in major incidents; from the unmatched transfusion of the 1880s to the functioning blood bank available to casualties of the second world war, Dr Glasgow looked at how transfusion is used in mass casualty events, be it train crashes, terrorist attacks or natural disasters the world over. He described the difficulties relating to blood product labelling, communication between supply and demand, miscommunication regarding public response to blood donation, and use of the most appropriate blood product. He warned delegates of being complacent in their attitude to the next potential threat and the subsequent strain on resources, including that of blood product requirements. Dr Glasgow concluded by demonstrating the innovative use of computer simulation modelling to aid planning for potential major incidents.

## Safety at terrorist incidents

The final three speakers of the day all addressed the theme theme of the risks posed to emergency services and medical providers in the line of duty. Having heard the first hand account of the Utoya shootings and the ongoing gunfire threatening the medical teams, the audience heard a succinct synopsis by Dr Julian Thompson, executive director of London Air Ambulance, of how to assess risk of personal threat as a first responder. Using open access medical literature, he presented a review of several international terrorist attacks, and the types of risks posed to rescuers; both those designed with malign intent, and those resulting from environmental factors.

Secondary explosive devices, as seen in Beslan, 2004, often involve timer devices or motion/pressure sensors for deployment. Reassuringly rare, of 36,000 bombings reported in the US, in only 4 were secondary explosive devices found. Advice included being aware of and performing 360 degree searches to identify such devices, avoiding bunching of rescuers, and, on recognition of a potential secondary explosive device, the rule of 4 C’s: Confirm, Clear, Cordon and Communicate. Small Arms fire aimed at the emergency services has been reported in Beslan, Israel 1996, the Mumbai attacks 2008, and Utoya Norway. Other risks include Chemical incidents, and Biological agent. The identification of biological agents being often delayed and difficult. Such Biological secondary threats can include contaminants from the penetration of foreign body and organic material / blood born disease. Mitigation of these risks include considering vaccination, universal precautions and post-exposure prophylaxis.

The diagnosis of exposure to the rare of radiological and nuclear threats is also often delayed and methods of management include increasing the distance and shielding from the source, remaining upwind, and reducing cross contamination. A particularly practical tip was for the medical responders to refrain from eating and drinking on scene. The combined effect of all these potential hazards to the emergency services are however less significant than the most common dangers which are environmental. The risk of structural collapse and inhalation of airborne toxins (dust, asbestos) has killed more responders than the rarer potential risks that we perhaps fear more. Detrimental psychological side effects such as post traumatic stress disorder and accompanying depression and alcohol abuse are also important to consider, especially in untrained helpers at scene.

## The terrorist chemical threat - lessons of the recent past

Dr Mark Byers spoke on the subject of the terrorist chemical threat and challenged the techniques and approach of many existing plans to manage incidents. He discussed the investment made in the development of hazardous area response teams (HART), decontamination suits and monitors but stressed that benefits of these are not entirely established. Practicalities such as the time taken for decontamination in mass casualty situations, and the reaction behaviour seen amongst the general public, (in Tokyo local hospitals were still being swamped by the worried well 40 days after the initial attack) were discussed openly, as well as the practical admission that emergency services must accept a certain degree of risk to do their job; it is impossible to create a risk free environment.

To address these issues the advice given was to conduct a risk assessment and empower the team on the ground with proper incident management training. This should ideally ensure rapid physician-led recognition and diagnosis of the incident type, and a practical system of triage for extrication and decontamination. The latter does not change the effects of the initial exposure, but does reduce further exposure and protect rescuers and infrastructure from contamination. From experience, any plan for communication needs a backup plan to cope with failure, and the advice given to the public must be practical. Dr Byers bought the day full circle, with reference to how the new specialist training in prehospital care, as laid out by Sir Keith Porter, is our opportunity to instil such practical skills in a new breed of specialists.

## Polonium - lessons from London - Dr Jim Down

The day was brought to a close with a candid account of the case of Alexander Litvinenko by Dr Jim Down, who was the ITU consultant managing his poisoning by Polonium 210 in London in 2006. Enlightening as it was to hear the details of the complicated difficult diagnosis, the courses of therapy undertaken, and the interplay of different coordinating governmental agencies, Dr Down also gave a personal and honest reflection on the lessons learnt in the case in particular with regards to the effect such a high profile case has on the hospital staff. The difficulty of maintaining confidentiality when there was potential for financial gain from leaks to the press, the paranoia and rumour generation within the wider staff that resulted from not being able to disseminate information openly, and the anxiety felt amongst exposed staff, led to some key lessons. Simple measures such as informing people there is no further news to give would have led to less general mistrust, and being aware of the competing agendas of different non-clinical agencies is important to hold in the balance.

Dr Byers’ thoughts on the notion of a level of risk acceptance were complimented by a reminder that we have a responsibility to both our patients and our colleagues, to act, reassure and communicate with their best interests in mind.

